# Artery and venous sinus occlusion image score (AVOIS): A novel method to evaluate occlusive cerebral arteries and venous diseases

**DOI:** 10.1111/cns.13689

**Published:** 2021-06-19

**Authors:** Zhimin Wu, Qinglin Feng, Mengqi Liu, Jie Li, Xiaochuan Sun, Quanhong Shi, Yan Zhan, Wei Dan, Bocheng Yang, Dinghao Zheng, Yulong Xia, Yanfeng Xie, Li Jiang

**Affiliations:** ^1^ Department of Neurosurgery The First Affiliated Hospital of Chongqing Medical University Chongqing China; ^2^ Department of Neurosurgery Chongqing University Three Gorges Hospital Chongqing China; ^3^ Department of Radiology The First Affiliated Hospital of Chongqing Medical University Chongqing China; ^4^ Institution of Intelligent Technology and Engineering Chongqing University of Science and Technology Chongqing China

**Keywords:** acute anterior circulation infarct (ACI), acute ischemic stroke (AIS), artery and venous occlusion image score (AVOIS), cerebral venous sinus thrombosis (CVST)

## Abstract

**Aim:**

To establish an artery and venous sinus occlusion image score (AVOIS) which is compatible in both cerebral arteries and venous system diseases.

**Methods:**

A total of 188 consecutive patients with the final diagnosis of anterior circulation infarct (ACI) and 56 consecutive patients with cerebral venous and sinus thrombosis (CVST) were retrospectively studied. The AVOIS was developed based on the severity of occlusive changes of main intracranial arteries and venous sinuses (present = 0, partial occlusion = 1, absent = 2), and divided into four groups (CVST group: 0, 1‐5, 6‐10, >10. ACI group: 0, 1‐5, 6‐10, >10) arbitrarily. A receiver operating characteristic (ROC) curve was applied to discover the sensitivity and specificity of AVOIS. The National Institutes of Health Stroke Scale (NIHSS), Clot Burden Score (CBS) were set as the reference. Logistic regression models were developed to adjust for baseline clinical variables and AVOIS. Length of hospital stay (LOS) was also evaluated using the Kaplan‐Meier estimator.

**Results:**

For the CVST group, a positive correlation between AVOIS and NIHSS was discovered (Spearman's ρ = 0.54, *p* < 0.001). For the ACI group, ROC showed relatively high sensitivity (84.8%) and specificity (81.8%). Besides, the probability of time to discharge was significantly different among the AVOIS subgroups as well (*p* < 0.001).

**Conclusion:**

The AVOIS can be used to evaluate the treatment of patients with acute stroke caused by cerebral venous sinus thrombosis and anterior circulation large vessel occlusion. It is a reliable and convenient method that may help prompt prognosis and guide the treatment of individual patients.

## Graphical abstract

A novel score system called the Artery and Venous Sinus Occlusion Image Score (AVOIS) was developed based on quantification analysis of the intracranial thrombus in both artery and venous sinus (Table 1). According to the CTA or MRA, anterior circulation and venous sinuses were allotted different scores (present = 0, partial occlusion = 1, absent = 2) by researchers. The total score on AVOIS in anterior circulation or venous sinus was accumulated to 14 points.

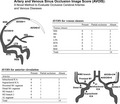

## 1 INTRODUCTION

Acute ischemic stroke (AIS) is an emergency with a critical time window of treatment.^1^, ^2^, ^3^ A classification tool for AIS must be reproducible, reliable, and suitable for the assessment of disease severity levels.^4^, ^5^ Previously, clinical rating instruments, such as the National Institutes of Health Stroke Scale (NIHSS),^6^ Boston Acute Stroke Imaging Scale (BASIS),^4^ Alberta Stroke Program Early CT Score (ASPECTS),^7^, ^8^ and Clot Burden Score (CBS) ^9^, ^10^ have been used to evaluate the early ischemic changes of the middle cerebral artery (MCA) and the brain parenchyma supplied by MCA in patients with stroke. However, the current methodologies have some shortcomings.^11^ For instance, NIHSS is limited in the ability to evaluate the effects of arterial occlusion treatment, and it provides little guidance on how to improve prompt and specific treatment.^4^, ^12^ CBS has been considered as an efficient evaluation tool for AIS of the anterior circulation,^13^ but its utilization is limited in the evaluation of vessels with partial filling defects.^4^

Furthermore, the above classification instruments focused specifically on patients with thrombotic stroke in cerebral arteries rather than thrombus formation in large veins (cerebral venous sinus). As far as we know, there is no relevant report on the scoring system which is based on the intracranial venous images and focuses on the evaluation of the patency of the intracranial veins. Although the intracranial venous system is as important as the arteries system, occlusion of cerebral veins, such as cerebral venous and sinus thrombosis (CVST), receives far less attention as compared with arterial stroke like acute anterior circulation infarct (ACI).

In this context, a novel classification instrument—Artery and Venous Occlusion Image Score (AVOIS)—was designed by quantification of cerebral arteries and venous sinuses changes on CTA/MRA images. To test the value of AVOIS in the evaluation of cerebral venous occlusion and artery disease, patients diagnosed with CVST and ACI were included in this study.

## 2 SUBJECTS AND METHODS

### 2.1 Patients

This is a retrospective study, and the inclusion met ethical standards approved by ethical committees of the First Affiliated Hospital of Chongqing Medical University. For patients who were conscious and cooperative, written informed consent was obtained from both patients and their legal guardians. For comatose patients, written informed consent was obtained from their legal guardians or their health care surrogates, and then from the patients themselves when they regained decision‐making capacity.

From 01/2019 to 01/2020, patients who had a final diagnosis of the CVST and ACI were included in the study. Non‐contrast CT (NCCT) or MRI was the default imaging modality in patients with suspected cerebral vascular disorder. The treating stroke neurologist decided to whether proceed to CTA/MRA and decided who was selected for endovascular therapy (EVT) based on CT/MRI characteristics. According to the chief cause of acute stroke, patients with ACI or CVST were naturally grouped. CTA or MRA was performed before and after treatment, and the initial NIHSS score and the follow‐up NIHSS were recorded in the electronic medical records. Other factors (ie, age, gender, and serum glucose) were extracted from medical records.

For the CVST group, inclusion criteria were given as: 1) age ranged 20‐80 years; 2) with acute disabling neurological deficits; 3) with significant symptoms (eg, headache and vomiting); 4) with the presence of an intraluminal filling defect on CTA. Exclusion criteria were given as: 1) with primary cerebral diseases (eg, intracranial space‐occupying lesions, encephalitis, psychosis, dementia, or psychosis, etc.); 2) with severe dysfunction of other organs or systems (such as serious respiratory and circulatory dysfunction, electrolyte disorders, etc.)

For the ACI group, inclusion criteria were given as: 1) age ranged 20‐80 years; 2) with acute disabling neurological deficits; 3) CTA was performed within 24h from symptom onset. Exclusion criteria were given as: 1) pre‐morbid modified Rankin scale (mRS) score >2; 2) with a history of AIS; 3) final diagnosis of transient ischemic attack (TIA); 4) nonischemic etiology. Clinical baseline variables including NIHSS and mRS score were routinely recorded in the patient records.

### 2.2 Imaging

Non‐contrast CT and CTA were performed on a 64‐multidetector row spiral CT machine (Somatom Sensation 64; Siemens Medical Systems). Contrast image acquisitions were obtained after a single bolus intravenous contrast injection of 90‐120 ml nonionic contrast media into an antecubital vein at 35 ml/s. All resource data were transferred to a dedicated workstation (Advantage for Windows; GE Medical Systems) for postprocessing.

MRI scanning data sets were obtained on a 1.5T MRI system (Sonata; Siemens) with echo‐planar capabilities. Imaging parameters were as follows: TR 5000 ms, TE 90 ms, FOV 22 × 22 cm, image matrix 128 × 128 pixels, section thickness 5 mm with a 1‐mm gap, 23 axial sections, and 5 signal intensity averages. The head MRA adopted 3D time‐flight‐technology with the flip angle 20°, TE 3.2 ms, TR 33.3 ms, 1 mm section thickness covering 6 cm, 512 × 512 matrix coding 22 cm field of view. Source images of MRA were reconstructed into maximum intensity projection (MIPS) views of the intracranial vasculature.

### 2.3 Assessment of thrombus burden

**FIGURE 1:**
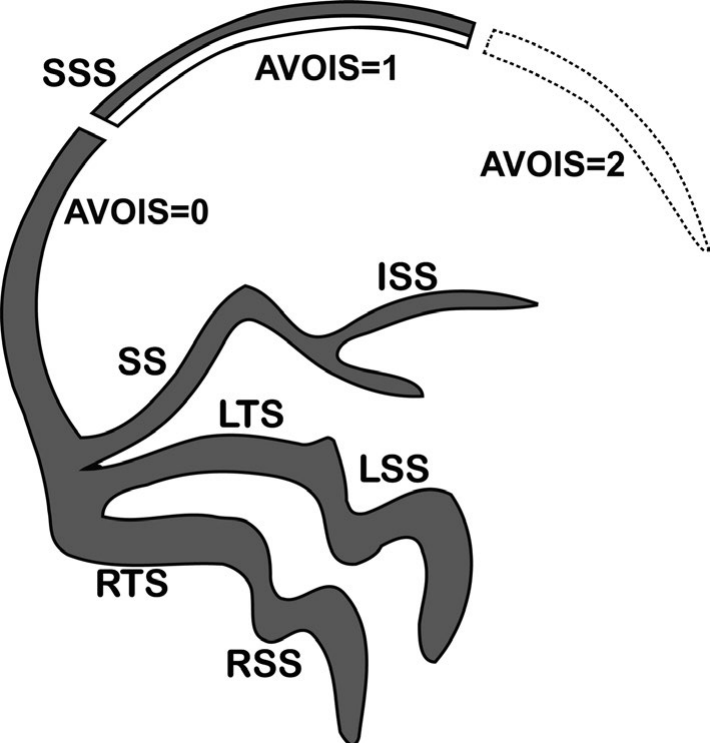
The artery and venous sinus occlusion image score (AVOIS) for cerebral venous sinus thrombosis (CVST). The venous sinuses were allotted different scores (present = 0, partial occlusion = 1, absent = 2) by quantifying the thrombus. SSS, superior sagittal sinus. ISS, inferior sagittal sinus. SS, straight sinus. LTS, left transverse sinus. RTS, right transverse sinus. LSS, left sigmoid sinus. RSS, right sigmoid sinus

**FIGURE 2:**
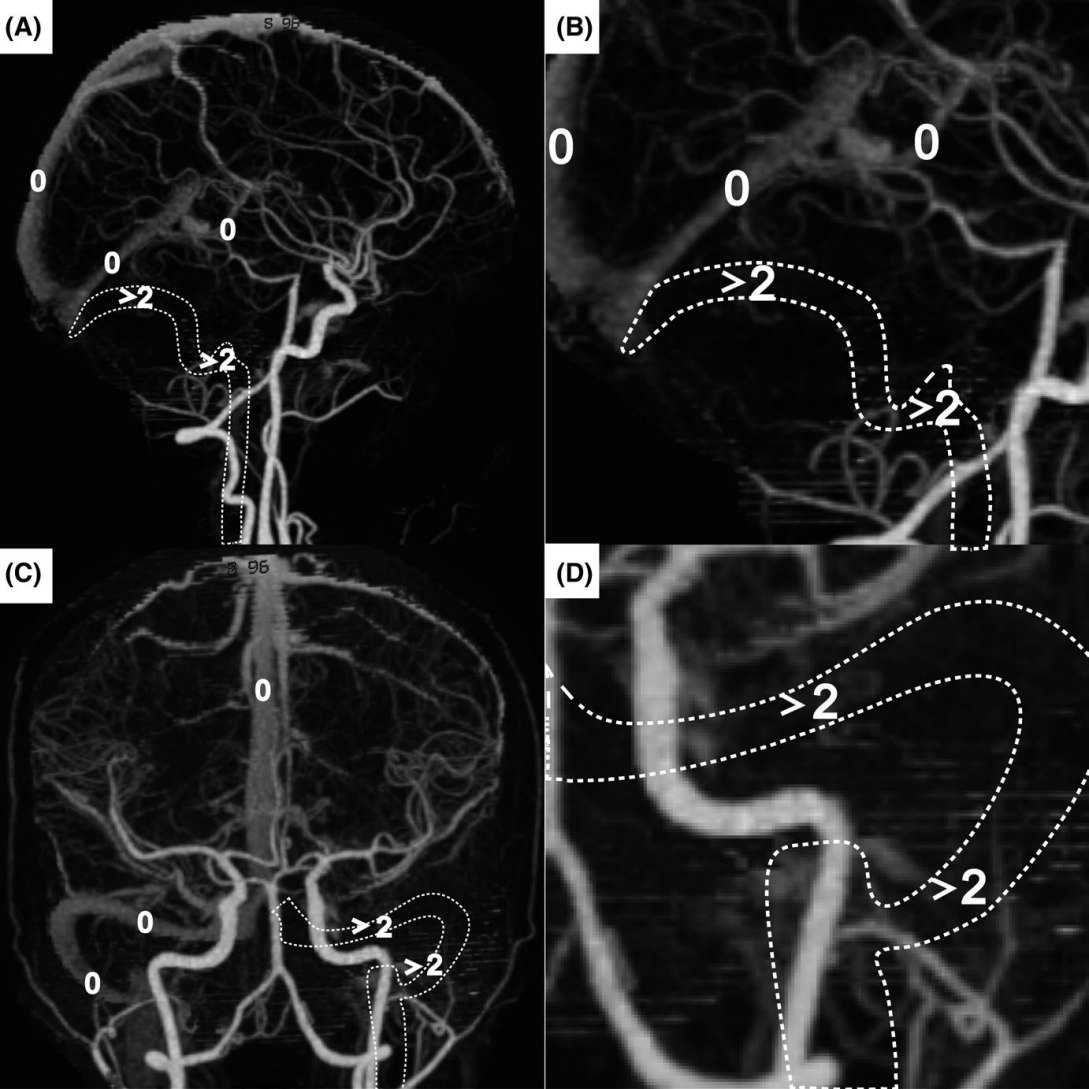
A maximum intensity projection (MIP) with manual removal of all skulls. A‐D, a patient with AVOIS = 4 (the absence of left transverse sinus and sigmoid sinus, two points for each segment, white arrowheads). AVOIS indicates Artery and Venous Sinus Occlusion Image Score

**FIGURE 3:**
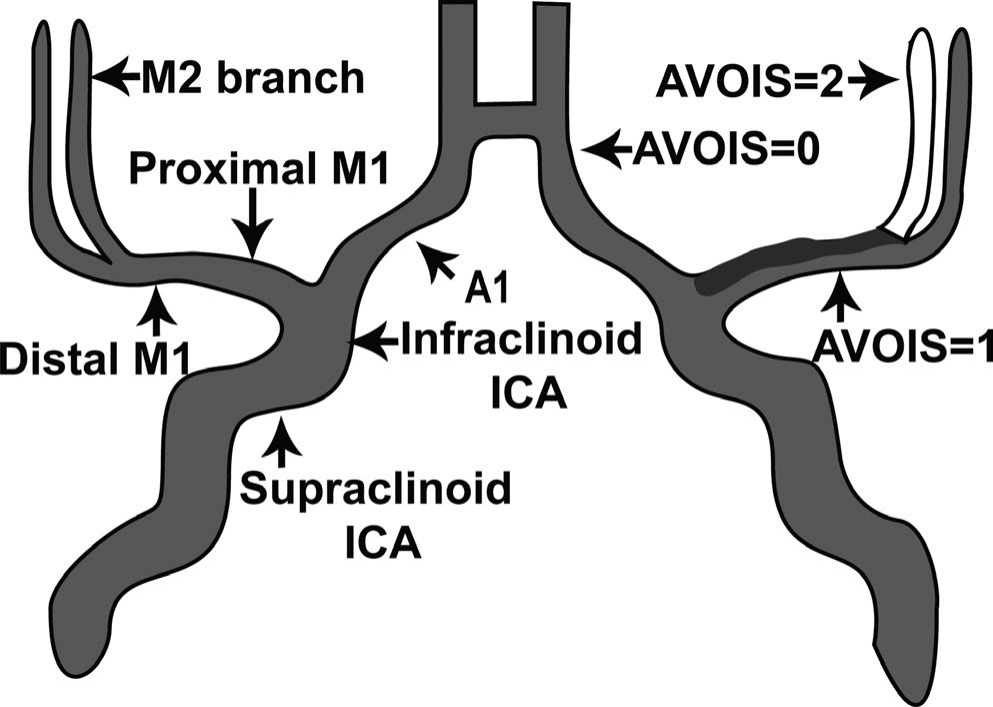
AVOIS for anterior circulation infarct (ACI). The anterior circulation was allotted different scores (present = 0, partial occlusion = 1, absent = 2) by quantifying the thrombus. ICA, internal carotid artery. M1, M1 segment of the middle cerebral artery. M2, M2 segment of the middle cerebral artery. A1, A1 segment of the anterior cerebral artery

**FIGURE 4:**
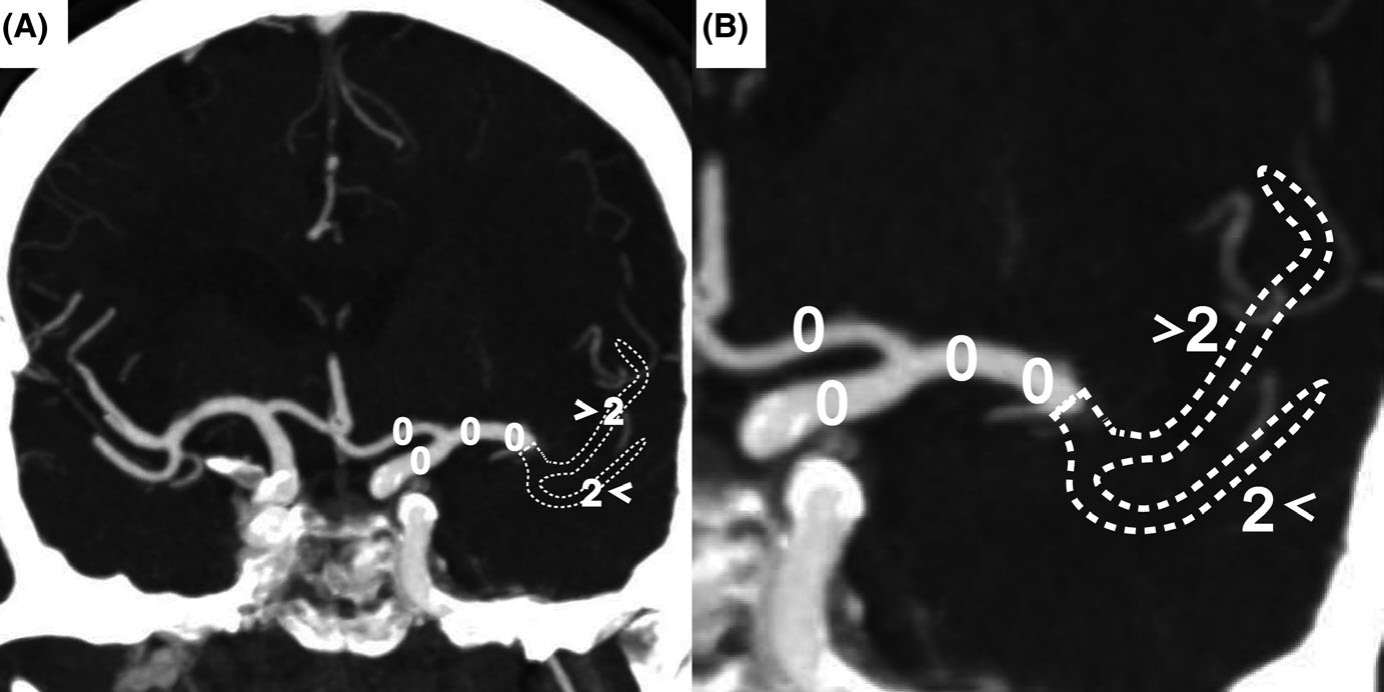
A maximum intensity projection (MIP) with manual removal of part of the sphenoid bone. A and B, a patient with AVOIS = 4 (the absence of left M2 branches, two points for each segment, white arrowheads). AVOIS indicates artery and venous sinus occlusion image score

A novel score system called the Artery and Venous Sinus Occlusion Image Score (AVOIS) was developed based on quantification analysis of the intracranial thrombus in both venous sinus and artery (Table 1, Figures 1, 2, 3, 4). According to the CTA or MRA, anterior circulation and venous sinuses were allotted different scores (present = 0, partial occlusion = 1, absent = 2) by two individual researchers through the double‐blind method. Except for the symptom side, they were blind to the patient's name, birth, and examination date. If there was a conflict between the two researchers, a third well‐trained researcher would review the images and give his interpretation. Then the final consensus was reached after a discussion. The total score on AVOIS in venous sinuses was accumulated to 14 points and was divided into four groups (0, 1‐5, 6‐10, >10) arbitrarily. Similar to the venous sinuses, the cumulative score in anterior circulation was 14 and divided into four groups (0, 1‐5, 6‐10 and >10) as well.

**TABLE 1:** Summary of the artery and venous sinus occlusion image score (AVOIS)

	Present	Partial occlusion	Absent
Venous sinuses			
SSS	0	1	2
ISS	0	1	2
SS	0	1	2
LTS	0	1	2
RTS	0	1	2
LSS	0	1	2
RSS	0	1	2
Total			14
Arteries			
Infraclinoid ICA	0	1	2
Supraclinoid ICA	0	1	2
Proximal M1 segment	0	1	2
Distal M1 segment	0	1	2
M2 branch 1	0	1	2
M2 branch 2	0	1	2
A1 segment	0	1	2
Total			14

Abbreviations: A1, A1 segment of the anterior cerebral artery; AVOIS, artery, and venous sinus occlusion image score; ICA, internal carotid artery; ISS, inferior sagittal sinus; LSS, left sigmoid sinus; LTS, left transverse sinus; M1, M1 segment of the middle cerebral artery; M2, M2 segment of the middle cerebral artery; RSS, right sigmoid sinus; RTS, right transverse sinus; SS, straight sinus; SSS, superior sagittal sinus.

### 2.4 Primary outcome measurement

The modified Rankin scale (mRS) score at 90 days was evaluated by a stroke neurologist in the stroke center in our hospital, who did not know the results of baseline NIHSS score, CT/CTA, MRI/MRA, or acute clinical events. Functional independence (favorable outcome) was defined as mRS score ≤2, unfavorable outcome as >2.

### 2.5 Statistical analysis

Statistical analyses were accomplished by IBM SPSS version 26.0 software (IBM SPSS Statistics). Data were presented using standard descriptive statistics. All data have been subject to tests for normality and data that do not exhibit a normal distribution was analyzed via a non‐parametric rank‐sum test (Kruskal‐Wallis Test). Multivariable logistic regression models were developed to adjust for baseline clinical variables and AVOIS in predicting clinical outcomes. Length of hospital stay (LOS) data was also evaluated using the Kaplan‐Meier estimator. *p*‐value <0.05 was required for significance. A receiver operating characteristic (ROC) curve was applied to discover the sensitivity and specificity of AVOIS in the diagnosis.

## 3 RESULTS

We identified 267 consecutive patients who performed CTA or MRA for clinically suspected acute ischemic stroke within 24 h from symptom onset. Among these, 56 patients had a final diagnosis of venous sinus system thrombosis, 188 patients had a final diagnosis of anterior circulation ischemic stroke, 13 patients had posterior circulation ischemic stroke. Another 10 patients were excluded as they were not accordant with the inclusion criteria for the unqualified pre‐mRS and NIHSS.

### 3.1 Cerebral venous and sinus thrombosis group

The clinical characteristics of 56 CVST patients are summarized in Table 2. Patients had a median age of 31 years [IQR 25‐40], of whom 27 (48%) were men. Fifty patients (89%) had a visible intracranial occlusion on CTV or MRV. The distribution of AVOIS values was skewed with a median AVOIS value of 7 (IQR 4‐10). Overall, the median baseline NIHSS score was 9 (IQR 6‐14), while the LOS was 18 (IQR 10‐23). Both NIHSS and LOS showed significant differences in different AVOIS subgroups (*p* < 0.05). A total of fifty‐two patients (93%) received anticoagulation or thrombectomy therapy (47 anticoagulation only, 1 i.v. thrombectomy only, 12 combined treatment). The different treatment methods and risk factors were not related to the value of AVOIS (*p* > 0.05).

**TABLE 2:** Baseline characteristics of CVST group

*N*	AVOIS 0	AVOIS 1‐5	AVOIS 6‐10	AVOIS >10	*p*
	6	22	18	10	
Age, median (IQR)	26 (23‐31)	25 (22‐28)	32 (26‐35)	31 (27‐43)	0.021^a^
Male, *n* (%)	4 (67)	8 (36)	10 (56)	5 (50)	0.452
NIHSS, median (IQR)	2 (1‐3)	5 (4‐9)	7 (5‐15)	14 (8‐19)	0.037 ^a^
LOS, median (IQR)	8 (5‐10)	10 (7‐15)	16 (12‐23)	21 (16‐24)	0.015 ^a^
Treatment, *n* (%)					
Anticoagulation only	3 (50)	20 (91)	16 (89)	8 (80)	0.528
I.v. thrombectomy only	0 (0)	1 (5)	0 (0)	0 (0)	0.673
Combined therapy	0 (0)	1 (5)	5 (28)	6 (60)	0.715
Risk factors, *n* (%)					
Arterial hypertension	1 (17)	2 (9)	4 (22)	3 (30)	0.325
Diabetes	0 (0)	3 (14)	1 (6)	1 (10)	0.461
Hypercholesterolemia	0 (0)	1 (5)	2 (6)	2 (20)	0.133
Smoking (current)	2 (33)	9 (41)	10 (56)	2 (20)	0.086
Coronary artery disease	0 (0)	2 (10)	1 (6)	0 (0)	0.181
Atrial fibrillation	0 (0)	1 (5)	3 (17)	1 (10)	0.540
History of TIA or stroke	0 (0)	2 (9)	4 (22)	3 (30)	0.212

Abbreviations: I.v., intravenous; IQR, interquartile range; LOS, length of hospital stays; NIHSS, National Institute of Health Stroke Scale; TIA, transient ischemic attack.

^a^ Non‐parametric rank‐sum test (Kruskal‐Wallis Test).

According to Table 3, age and NIHSS score were the risk factors in the prediction of unfavorable prognosis (age, *p* < 0.05, OR 1.03, CI_95_ 0.024. NIHSS, *p* < 0.05, OR 1.21, CI_95_ 0.007). To further test the validity and reliability of AVOIS in the prediction of poor outcomes, we made a comparison in the subgroups of AVOIS. AVOIS in the CVST group showed highly significant differences in various subgroups. Interestingly, the higher clot burden was accompanied by the higher OR and the poorer clinic outcome in both favorable and unfavorable groups. When the AVOIS score increased, the risk of acquiring an unfavorable outcome (mRS > 2) increased accordingly (Table 4).

**TABLE 3:** The outcome of 56 patients with CVST according to categorized mRS at 90 days

Parameter^a^	mRS ≤ 2	mRS > 2	Logistic regression analysis		
	*n* = 141	*n* = 47	OR	CI_95_	*p*
Age	28 (22‐39)	40 (33‐52)	1.03^b^	1.01‐1.27	0.024
AVOIS	5 (3‐6)	7 (3‐9)	1.97^c^	1.23‐4.88	0.006
NIHSS	6 (2‐9)	13 (10‐18)	1.21^c^	1.36‐2.55	0.007

Note

*p* < 0.05 for significant.

Abbreviations: CI_95_, 95% confidence interval; mRS, modified rankin scale; NIHSS, the National institutes of health stroke scale; OR, odds ratio.

^a^ median (interquartile range).

^b^ per year increase.

^c^ per point increase.

**TABLE 4:** The logistic regression analysis for the outcome of CVST group according to the grouped AVOIS

AVOIS	mRS ≤ 2			mRS > 2		
	OR	CI_95_	*p*	OR	CI_95_	*p*
0	1.00			1.00		
1‐5	0.57	(0.49‐0.83)	0.069	2.03	(0.85‐6.78)	0.077
6‐10	0.43	(0.33‐0.76)	0.024	4.51	(0.76‐7.28)	0.041
> 0	0.28	(0.10‐0.45)	0.002	5.98	(1.04‐17.31)	0.008

Note

*p* < 0.05 for significant.

Abbreviations: CI_95_, 95% confidence interval; mRS, modified Rankin scale; OR, odd ratio.

**FIGURE 5:**
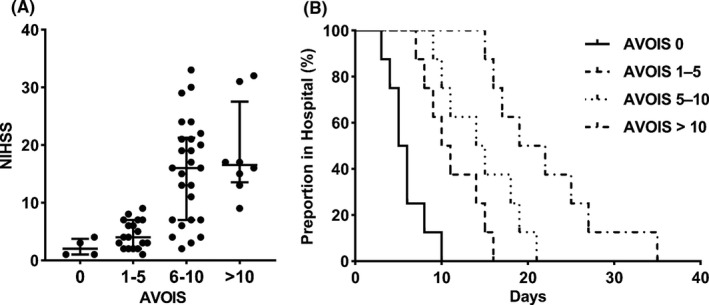
A, The National Institutes of Health Stroke Scale (NIHSS) score for categorized AVOIS subgroups. Patients with higher AVOIS had higher NIHSS score (range 0‐42). B, Kaplan‐Meier curve of time to discharge. The Kaplan‐Meier graph depicts the probability of discharge from the hospital in days for patients evaluated by AVOIS. Patients with lower AVOIS may get better effects within relatively short LOS, whereas the clinical outcome of patients with higher AVOIS may be unfavorable

In the CVST group, patients with higher AVOIS (ie, higher thrombus burden) had higher baseline NIHSS scores (Figure 5A). There was a highly significant positive correlation of AVOIS with NIHSS scores (Spearman's ρ = 0.56, *p* < 0.001). We applied the Kaplan‐Meier estimator to evaluate the LOS data (Figure 5B). This is a kind of analysis commonly used to test the curative effect such as survival and had been applied to estimate the probability of time to discharge from the hospital.^4^ The probability of time to discharge was significantly different among the subgroups of CVST (*p* < 0.001). It was shorter for patients with AVOIS = 0 compared with the other subgroups, suggesting patients with lower AVOIS may get better effects within relatively short LOS, whereas the clinical outcome of patients with higher AVOIS may be unfavorable. Overall, highly significant differences (*p* < 0.001) in CVST subgroups were found.

### 3.2 Anterior circulation infarct group

This report describes 188 ACI patients with a median age of 68 years [interquartile range (IQR) 52‐71], of whom 101 (54%) were men (Table S1). The logistic regression analysis showed significant differences existed in age, CBS, AVOIS, ASPECTS, and NIHSS (*p* < 0.05). The outcome of 141 in 188 patients was favorable (mRS ≤2), while the number of unfavorable (mRS > 2) outcomes was 47. Among these, the OR of AVOIS was 1.78 (*p* < 0.05), which suggested the clinical outcome of AIS patients in this study would get poorer per point increase (Table S2). In various subgroups, AVOIS in the ACI group showed highly significant differences. (Table S3).

**FIGURE 6:**
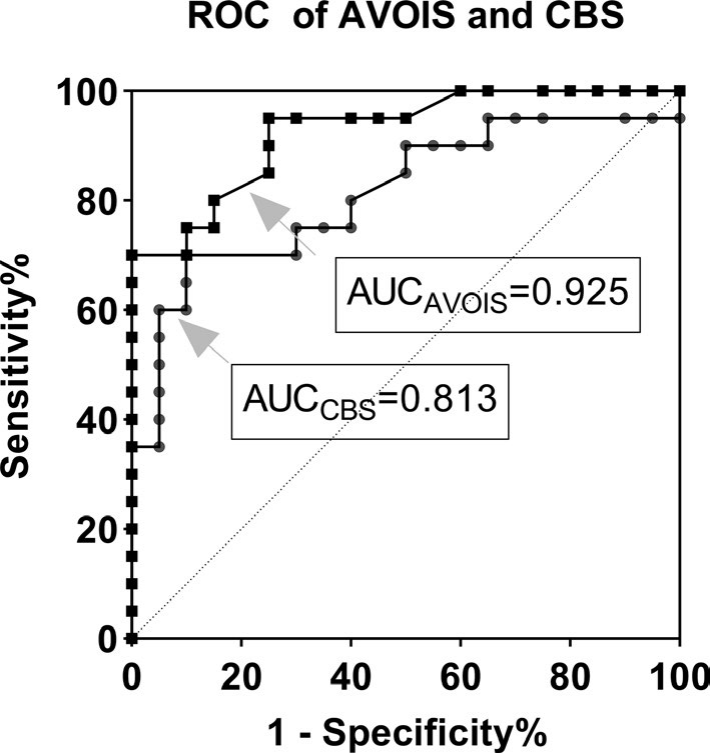
The receiver operating characteristic curve (ROC) of AVOIS and CBS. The area under the curve (AUC) of AVOIS was 0.925, which was larger than the area of the CBS (AUC_CBS_ = 0.813). The best cut‐off point of AVOIS was 5.5, the sensitivity 84.8%, and the specificity 81.8% (95% CI: 0.822‐0.950)

To assess whether AVOIS was superior to CBS in the diagnosis of ACI, ROC was utilized for testing the sensitivity and specificity (Figure 6). In the ACI group, AVOIS showed relatively high sensitivity (84.8%) and specificity (81.8%), and the best cutoff point was 5.5. The area under the curve (AUC) of AVOIS was 0.925, which was larger than the area of the CBS (AUC_CBS_ = 0.813).

## 4 DISCUSSION

In the present study, a novel neuroimaging‐based stroke score system called AVOIS has been developed. After being tested in CVST and ACI patients and compared with other image scoring tools, we found that AVOIS was an effective method that was compatible in both the cerebral venous system and the arteries. The LOS and prognosis could be also predicted by AVOIS in both CVST and ACI groups. For CVST patients, AVOIS provided an additional novel evaluation method for thrombus formation in large cerebral veins (venous sinus) and was highly effective and accurate in predicting poor outcomes. In the ACI group, AVOIS was also shown to be of important practical value as it is simple to use and can yield reliable information for the evaluation of thrombotic stroke in cerebral arteries.

One of the main purposes of this study was therefore to find a reliable method to evaluate the intracranial venous system. By testing in CVST patients, we found the availability of AVOIS in the assessment of occlusive intracranial veins. Although patients with CVST have a favorable prognosis,^14^ a few patients have extremely variable and nonspecific clinical manifestations, which make the diagnosis of CVST not easy and mislead the subsequent treatment.^15^ Therefore, it would be of essence to establish a rapid and clinically applicable evaluation tool for CVST. In the absence of a special image classification tool, this cerebrovascular disorder depends mainly on some symptom‐based clinical evaluation instruments.^16^ Currently, the NIHSS is generally acknowledged as the most validated and the most widely used clinical rating instrument with scores ranging from 0 to 42.^17^ In this research, we set the NIHSS as the reference to study the AVOIS and found NIHSS scores increased with the increasing primary AVOIS stage. A positive correlation between AVOIS and NIHSS was observed (Spearman correlation coefficient, 0.61; *p* < 0.001), which indicated that this novel scoring tool may have the potential to grade the severity of CVST and benefit patients. As compared with NIHSS, one of the main advantages of AVOIS is not dependent on symptoms and invasive angiography, so it is easy to obtain when trying to determine the best treatment for a single patient. For patients who are unable to cooperate with the physical examination, AVOIS provides a valuable supplement to traditional evaluation approaches. Besides, in the evaluation of prognosis, the Kaplan‐Meier graph showed the hospital LOS for patients with different AVOIS differed significantly in the four subgroups, demonstrating the LOS and prognosis could be predicted by AVOIS in the CVST patients. For example, the LOS of patients with AVOIS > 10 was almost three times as high as that of AVOIS = 0. Also, the logistic regression analysis showed that AVOIS was one of the risk factors for unfavorable outcomes at 3 months (mRS > 2), and a higher AVOIS was strongly associated with a poor prognosis. All these findings suggested that AVOIS might have potential clinical application in the diagnosis and follow‐up of CVST. Based on this analysis, we speculated that patients with lower AVOIS scores could more easily achieve a better prognosis.

Another purpose of this study was to test the availability of AVOIS in the assessment of unilateral occlusive changes in the major cerebral arteries. As we know, ASPECTS is based on brain parenchymal damage on NCCT,^7^ and NIHSS is mainly based on a standard neurological examination.^18^ CBS and BATMAN were designed for anterior circulation and posterior circulation respectively and were both angiography‐based scoring systems.^13^, ^19^ However, CBS has a minor flaw in that partial filling defects in anterior circulation were rated as patent when evaluating,^13^ which may be unreasonable because that may lead to biases in the evaluation of partial occlusion of the blood vessel. Likewise, we noticed similar potential problems in the scoring tool of the vertebra basilar system (BATMAN). In AVOIS, partial occlusion (ie, partially present, or congenital stenosis) was allotted one point, while complete occlusion and no occlusion were allotted two and zero separately. This is one of the main differences between CBS and AVOIS. To gain more insight in the diagnosis of ACI, we performed a ROC analysis and found AVOIS had a higher AUC‐ROC (0.925) as compared to CBS (0.813), demonstrating this novel instrument was highly sensitive, specific, and accurate in diagnosing.

In conclusion, we believe that the above findings on AVOIS are novel and might have clinical significance. First, as a classification tool of stroke, AVOIS has been proven to be compatible in both cerebral venous system and arteries. Second, in cases in which cerebral venous sinus thrombosis and infraction were suspected, AVOIS may aid in diagnosis and decisions about disposition due to its accuracy, convenience, and simplicity. Finally, our findings in this study suggested that AVOIS has potential clinical application in the follow‐up of occlusive cerebrovascular diseases.

However, we must acknowledge there are several methodological shortcomings of the AVOIS in the assessment. First, the thrombosis in deep cerebral veins, such as cavernous sinus, inferior and superior petrosal sinus, cannot be evaluated according to this version of the image score. Second, known contrast agent allergy has limited the clinical application of AVOIS in some patients. Third, the LOS in this report could be affected by various factors such as pneumonia, urinary tract infection, sepsis, and complication with other system diseases, which may influence the total hospital stay time. What's more, this is a single‐center and small sample research and needs further study to confirm the validity of AVOIS.

## 5 CONCLUSION

The AVOIS provides a novel accurate semi‐quantity evaluation method to evaluate occlusive cerebral arteries and venous diseases and may help guide the treatment of individual patients and predict prognosis.

## CONFLICT OF INTEREST

The authors have no conflicts of interest to declare.

## AUTHOR CONTRIBUTIONS

All authors contributed to the study conception. This study was designed and managed by Yanfeng Xie and Li Jiang. Material preparation, data collection, and analysis were performed by Zhimin Wu, Qinglin Feng, Mengqi Liu, Yanfeng Xie, and Jie Li. The first draft of the manuscript was written by Zhimin Wu, Qinglin Feng, and Li Jiang. All authors commented on previous versions of the manuscript. All authors read and approved the final manuscript.

## CONSENT TO PARTICIPATE

Informed consent was obtained from all individual participants included in the study.

## CONSENT FOR PUBLICATION

Written consent for publication was obtained from the parents or legal guardians of patients.

## Supporting information

Table S1‐S3

## Data Availability

Data available on request due to privacy/ethical restrictions.
