# Supplementary material for: Artery and venous sinus occlusion image score (AVOIS): A novel method to evaluate occlusive cerebral arteries and venous diseases
Source: CNS Neurosci Ther. 2021 Jun 19;27(9):1077–84. doi: 10.1111/cns.13689 (PMC8339536; doi:10.1111/cns.13689)
Supplement: Supplementary file 1 — Table S1‐S3 [file CNS-27-1077-s001.docx]

| Table 1. Baseline characteristics of the anterior circulation infarct (ACI) group according to the categorized artery and venous sinus occlusion image score (AVOIS) | | | | | |
| --- | --- | --- | --- | --- | --- |
|  | AVOIS  0 | AVOIS  1~5 | AVOIS  6~10 | AVOIS  >10 |  |
| *N* | 86 | 65 | 22 | 15 | *P* |
| Age, median (IQR) | 75 (58–69) | 71(61–78) | 69 (61–77) | 74 (51–83) | 0.008* |
| Male, *n* (%) | 51 (59) | 30 (46) | 14 (64) | 6 (40) | 0.132 |
| CBS, median (IQR) | 10 (10) | 8 (4–9) | 6 (5–8) | 5 (3–7) | 0.077* |
| ASPECTS, median (IQR) | 10 (8–10) | 7 (5–9) | 7 (5–9) | 5 (3–8.5) | 0.001* |
| NIHSS, median (IQR) | 7 (6–11) | 12 (8–18) | 15 (11–19) | 18 (15–20) | 0.002* |
| LOS, median (IQR) | 11 (9–14) | 15 (13–21) | 21 (14–26) | 24 (13–29) | 0.001* |
| Any thrombolysis, *n* (%) |  |  |  |  |  |
| I.v. tPA only | 25 (29) | 28 (43) | 15 (68) | 12 (80) | 0.368 |
| I.a. therapy only | 0 (0) | 14 (22) | 9 (41) | 6 (40) | 0.143 |
| Combined i.v.–i.a. therapy | 0 (0) | 4 (6) | 7 (32) | 3 (20) | 0.325 |
| Risk factors, *n* (%) |  |  |  |  |  |
| Arterial hypertension | 56 (65) | 38 (58) | 17 (77) | 10 (67) | 0.157 |
| Diabetes | 16 (19) | 17 (26) | 8 (36) | 3 (20) | 0.254 |
| Hypercholesterolemia | 25 (29) | 14 (22) | 7 (32) | 4 (27) | 0.082 |
| Smoking (current) | 19 (22) | 10 (15) | 8 (36) | 6 (40) | 0.376 |
| Coronary artery disease | 11 (13) | 9 (14) | 7 (32) | 3 (20) | 0.841 |
| Atrial fibrillation | 9 (10) | 11 (17) | 3 (14) | 3 (20) | 0.540 |
| History of TIA or stroke | 18 (21) | 19 (29) | 7 (32) | 4 (27) | 0.212 |
| TOAST, *n* (%) |  |  |  |  | 0.051 |
| Large artery | 17 (20) | 20 (31) | 4 (18) | 6 (40) |  |
| Cardioembolic | 30 (35) | 18 (28) | 9 (41) | 7 (47) |  |
| Small vessel | 26 (30) | 6 (9) | 3 (14) | 1 (7) |  |
| Other determined | 5 (6) | 2 (3) | 4 (18) | 0 (0) |  |
| Undetermined | 8 (9) | 19 (29) | 2 (9) | 1 (7) |  |

Note: * Non-parametric rank-sum test (Kruskal-Wallis Test). IQR, interquartile range; NIHSS, National Institute of Health Stroke Scale; LOS, length of hospital stays; i.v., intravenous; i.a., intra-arterial; tPA, tissue plasmogen activator; TIA, transient ischemic attack; TOAST, Trial of Org 10172 in Acute Stroke Treatment.

A total of 188 patients had a final diagnosis of anterior circulation ischemic stroke. One hundred and two patients (54%) had a visible intracranial occlusion on CTA or MRA. The distribution of AVOIS values was skewed with a median AVOIS value of 5 (IQR 3–8). Overall, the median baseline ASPECTS and NIHSS scores were 7 (IQR 4–9) and 12 (IQR 6–15), while the LOS was 22 (IQR 17–25). A total of 109 patients (58.0%) received i.v. thrombolysis or mechanical i.a. therapy (80 i.v. tPA only, 29 i.a. treatment only, 14 combined i.v.–i.a. treatment,). The different treatment methods in the four groups showed no significance (*P*>0.05). Risk factors such as hypertension, diabetes, hypercholesterolemia, as well as TIA or stroke history seem to be not related to the value of AVOIS (*P*>0.05) in this study. However, the proportion of TOAST subtypes in various AVOIS groups showed a significant difference (*P*<0.05).

| Table 2. The outcome of 188 patients with anterior circulation infarct (ACI) according to the categorized mRS at 90 days | | | | | | |
| --- | --- | --- | --- | --- | --- | --- |
|  | mRS≤2 | mRS>2 |  | Logistic regression analysis | | |
| Parameter* | n=141 | n=47 |  | OR | CI_95_ | *P* |
| Age | 69 (45–73) | 71 (51–68) |  | 1.21§ | 1.06–1.34 | 0.009 |
| CBS | 8 (6–9) | 6 (4–7) |  | 2.10¶ | 0.65–7.13 | 0.032 |
| AVOIS | 4 (2–6) | 7 (5–9) |  | 1.78† | 0.89–5.61 | 0.011 |
| ASPECTS | 7 (5–8) | 5 (3–6) |  | 1.33¶ | 0.42–6.77 | 0.043 |
| NIHSS | 8 (5–12) | 11 (9–16) |  | 1.04† | 0.73–4.68 | 0.026 |

Note: mRS, modified Rankin scale; OR, odd ratio; CI_95_, 95% confidence interval; *P*<0.05 for significant; *median (interquartile range); §per year increase; ¶per point subtract; †per point increase.

| Table 3. The logistic regression analysis for outcome according to the grouped clot burden score (CBS) and artery and venous sinus occlusion image score (AVOIS) | | | | | | | |
| --- | --- | --- | --- | --- | --- | --- | --- |
|  | mRS ≤ 2 | | |  | mRS > 2 | | |
| Parameter | OR | CI_95_ | *P* |  | OR | CI_95_ | *P* |
| CBS |  |  |  |  |  |  |  |
| 10 | 1.00 |  |  |  | 1.00 |  |  |
| 8–9 | 0.51 | (0.18–0.76) | 0.032 |  | 2.01 | (0.72–5.48) | 0.015 |
| 6–7 | 0.34 | (0.13–0.45) | 0.017 |  | 3.54 | (1.21–6.59) | 0.043 |
| < 6 | 0.11 | (0.10–0.42) | <0.001 |  | 9.56 | (5.71–28.14) | <0.001 |
| AVOIS |  |  |  |  |  |  |  |
| 0 | 1.00 |  |  |  | 1.00 |  |  |
| 1–5 | 0.69 | (0.64–0.95) | 0.024 |  | 1.73 | (0.79–7.21) | 0.245 |
| 6–10 | 0.37 | (0.42–0.77) | 0.013 |  | 3.61 | (0.46–8.04) | 0.036 |
| > 10 | 0.15 | (0.15–0.52) | 0.006 |  | 6.11 | (2.33–19.21) | <0.001 |

Note: mRS, modified Rankin scale; OR, odd ratio; CI_95_, 95% confidence interval; *P*<0.05 for significant.
